# Supplementary material for: The MiR-320 Family Is Strongly Downregulated in Patients with COVID-19 Induced Severe Respiratory Failure
Source: Int J Mol Sci. 2021 Sep 26;22(19):10351. doi: 10.3390/ijms221910351 (PMC8508658; doi:10.3390/ijms221910351)
Supplement: Supplementary file 1 [file ijms-22-10351-s001.zip › ijms-1375663-supplementary/Table S1_Figure S1.pdf]

Table S1.

KEGG Pathway	p-value	#Genes	#miRNAs
Hippo signaling pathway	8,41E-06	20	3
TGF-β signaling pathway	5,16E-02	13	2
Adherens junction	5,62E-01	12	3
Glioma	0.0006464415	11	2
Transcriptional mis-regulation in cancer	0.001225354	26	1
Viral carcinogenesis	0.003771688	27	1
Estrogen signaling pathway	0.01097114	15	1
Colorectal cancer	0.01129309	11	1
Endometrial cancer	0.01132072	6	1
Pancreatic cancer	0.01234261	11	1
Signaling pathways regulating pluripotency of stem cells	0.0231285	18	1
Pathways in cancer	0.0329894	42	1
Chronic myeloid leukemia	0.03852175	14	1
Cocaine addiction	0.1069309	6	1
Sulfur relay system	0.1277975	2	1
Long-term depression	0.1390326	9	1
Spliceosome	0.2956839	18	1

Figure S1.

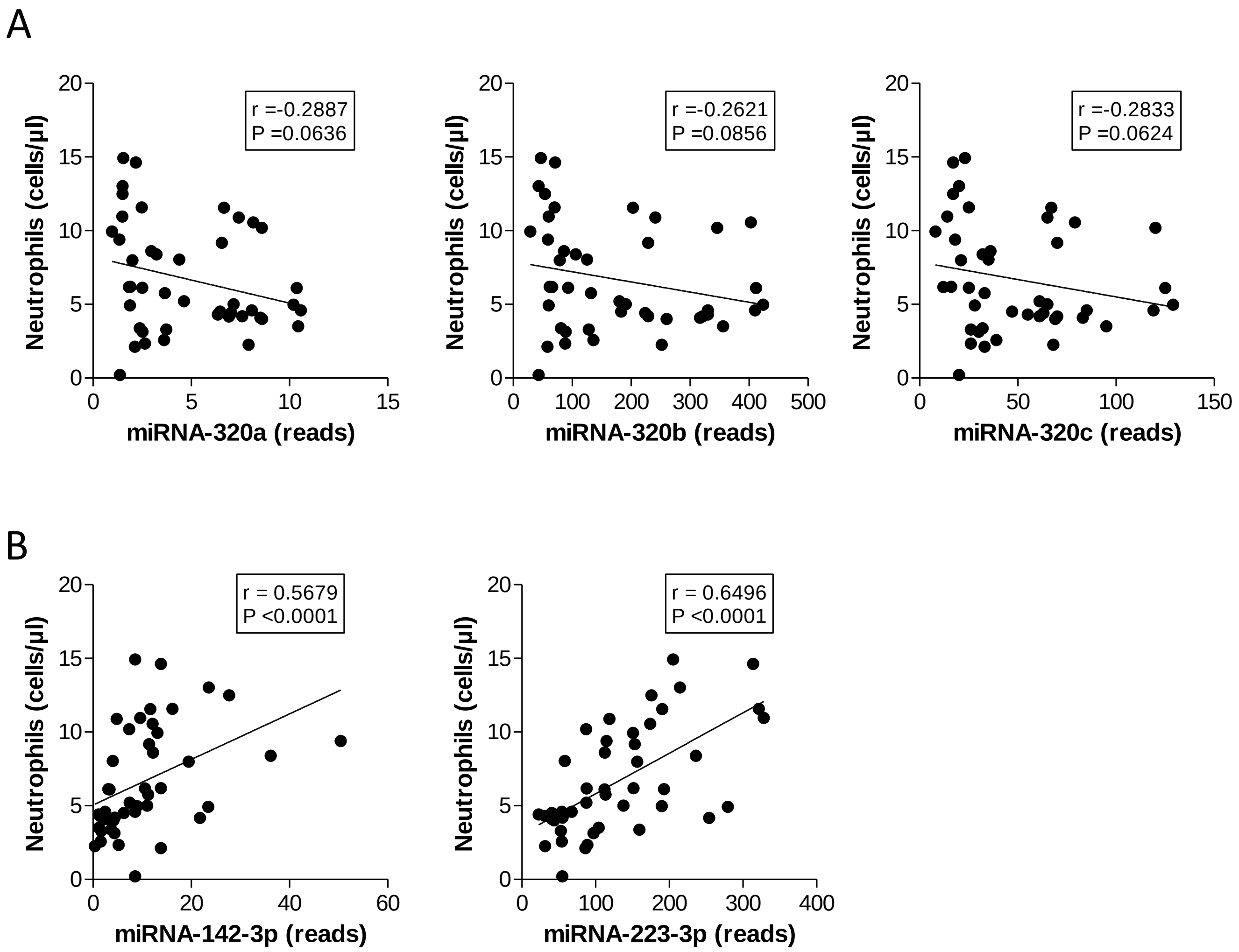
A
r =-0.2887
P =0.0636
Neutrophils (cells/µl)
miRNA-320a (reads)
r =-0.2621
P =0.0856
Neutrophils (cells/µl)
miRNA-320b (reads)
r =-0.2833
P =0.0624
Neutrophils (cells/µl)
miRNA-320c (reads)
B
r = 0.5679
P <0.0001
Neutrophils (cells/µl)
miRNA-142-3p (reads)
r = 0.6496
P <0.0001
Neutrophils (cells/µl)
miRNA-223-3p (reads)
