# Supplementary material for: The MiR-320 Family Is Strongly Downregulated in Patients with COVID-19 Induced Severe Respiratory Failure
Source: Int J Mol Sci. 2021 Sep 26;22(19):10351. doi: 10.3390/ijms221910351 (PMC8508658; doi:10.3390/ijms221910351)
Supplement: Supplementary file 1 [file ijms-22-10351-s001.zip › ijms-1375663-supplementary/Table S2.pdf]

Table S2

Significantly deregulated miRNA in COVID-19 patients with severe respiratory failure (> day7)

Int_V2	baseMean	log2FoldChange	lfcSE	pvalue	padj
hsa-miR-4747-3p	4,55764879	-6,049793888	0,791009026	2,04E-14	2,45E-12
hsa-miR-4429	4,38568047	-6,026816409	0,76956777	4,82E-15	8,12E-13
hsa-miR-6729-3p	4,37458866	-5,982705119	0,833046254	6,88E-13	4,14E-11
hsa-miR-1908-5p	4,39011405	-5,72873334	1,444625879	7,32E-05	0,000346856
hsa-miR-4707-5p	3,23759672	-5,602578612	0,865367714	9,53E-11	4,18E-09
hsa-miR-6766-3p	3,11481399	-5,531317454	0,854042279	9,38E-11	4,18E-09
hsa-miR-4508	12,1812401	-5,492177963	1,003413235	4,41E-08	7,04E-07
hsa-miR-6763-3p	2,64109076	-5,288071544	1,070616328	7,84E-07	7,34E-06
hsa_piR_020439/gb,	2,59814384	-5,275792499	1,874577864	0,004886996	0,012343115
hsa_piR_017936/gb,	2,11759791	-4,968564487	0,893884133	2,72E-08	4,98E-07
hsa-miR-6724-5p	2,06877582	-4,962582324	1,042360855	1,93E-06	1,55E-05
hsa-miR-1343-3p	6,05148598	-4,77202489	0,870297967	4,18E-08	6,90E-07
hsa-miR-7110-5p	1,62833257	-4,602570628	1,09328291	2,56E-05	0,000141548
hsa-miR-4728-3p	1,63035032	-4,558164615	1,195850443	0,000138042	0,000594823
hsa-miR-1238-3p	3,5536057	-4,484441631	0,828464268	6,20E-08	9,00E-07
hsa-miR-6797-3p	2,63303729	-4,35160212	1,013407321	1,75E-05	0,000102379
hsa-miR-3150a-3p	1,31482098	-4,268466877	1,34031722	0,001449196	0,004519344
hsa_piR_005021/gb,	1,24358408	-4,255684684	1,372448337	0,001930001	0,00557962
hsa-miR-6771-5p	1,1854338	-4,145628297	1,354158959	0,002203013	0,006183122
hsa-miR-6804-3p	2,17492005	-4,068709009	0,846283995	1,53E-06	1,30E-05
hsa-miR-1266-5p	1,19243869	-3,80970294	1,450042273	0,008606422	0,020018252
hsa-miR-3127-5p	2,36483311	-3,313272502	0,844879731	8,80E-05	0,000398213
hsa-miR-616-3p	3,70792222	-3,241811981	0,718697414	6,46E-06	4,19E-05
hsa-miR-2276-3p	2,41413308	-3,160528143	1,029593802	0,00214285	0,00603438
hsa-miR-320d	10,2468535	-3,00126817	0,475255839	2,70E-10	1,03E-08
hsa-miR-4473	1,0750181	-2,966636188	1,299291414	0,022414329	0,045919381
hsa-miR-3656	19,3526143	-2,937104047	0,467140064	3,23E-10	1,18E-08
hsa-miR-3945	2,31505376	-2,927686406	1,005498583	0,00359495	0,009452045
hsa_piR_020548/gb,	3,73972464	-2,790055467	0,737298022	0,000154227	0,000649296
hsa-miR-6739-3p	2,42825961	-2,782344263	1,010815479	0,005912881	0,014557443
hsa-miR-320e	4,26657227	-2,70394799	0,572502058	2,32E-06	1,79E-05
hsa-miR-320a	5291,36295	-2,673358529	0,215071495	1,79E-35	1,51E-32
hsa-miR-6882-3p	14,2943233	-2,652783273	0,489910086	6,13E-08	9,00E-07
hsa-miR-320b	181,562807	-2,649546871	0,263845689	9,96E-24	4,19E-21
hsa-miR-4433b-5p	107,195811	-2,592587778	0,623575442	3,22E-05	0,000171376
hsa-miR-423-5p	19364,4278	-2,484521345	0,341096436	3,24E-13	2,28E-11
hsa-let-7b-5p	15344,7489	-2,405269952	0,327019707	1,91E-13	1,78E-11
hsa-miR-4646-3p	28,1123356	-2,36419473	0,45885069	2,57E-07	2,89E-06
hsa-miR-1299	16,1230562	-2,361236421	0,826229829	0,004265293	0,010982803
hsa-miR-320c	47,1043346	-2,34672353	0,285292363	1,94E-16	5,45E-14
hsa-miR-1306-3p	10,3386013	-2,329662474	0,49691454	2,76E-06	2,07E-05
hsa-miR-1306-5p	406,148243	-2,229005706	0,43344055	2,71E-07	3,00E-06
hsa-miR-4326	178,220117	-2,171684808	0,372565498	5,58E-09	1,17E-07
hsa-miR-6726-3p	23,3114181	-2,16641282	0,385063226	1,84E-08	3,53E-07
hsa-miR-5010-3p	101,860885	-2,164509758	0,299790661	5,20E-13	3,37E-11
hsa-miR-4732-5p	238,095366	-2,133851246	0,305781508	2,99E-12	1,68E-10

hsa-miR-4755-5p	12,1371573	-2,086658299	0,500397713	3,05E-05	0,000164406
hsa-miR-6793-3p	8,62475254	-2,082802877	0,463597039	7,03E-06	4,49E-05
hsa-miR-636	275,075693	-2,071242857	0,355194793	5,50E-09	1,17E-07
hsa-miR-6777-3p	20,0768737	-2,065442012	0,427705115	1,37E-06	1,22E-05
hsa-miR-6820-3p	11,6743438	-2,047148965	0,42817509	1,74E-06	1,44E-05
hsa-miR-4685-3p	143,841218	-2,021301521	0,388687503	1,99E-07	2,41E-06
hsa-miR-4257	6,04091768	-2,014834637	0,577368826	0,000483592	0,001747574
hsa_piR_016659/gb,	47265,0932	-2,012145136	0,345183517	5,57E-09	1,17E-07
hsa-miR-3135b	50,9644896	-2,005247924	0,437684628	4,62E-06	3,16E-05
hsa-miR-4742-3p	235,285451	-1,997179603	0,43737088	4,96E-06	3,34E-05
hsa_piR_016658/gb,	28036,7396	-1,972810383	0,329370728	2,10E-09	5,54E-08
hsa-miR-6750-3p	10,9701095	-1,968631074	0,446503507	1,04E-05	6,29E-05
hsa-miR-584-3p	16,7757204	-1,95781246	0,478038586	4,21E-05	0,000217596
hsa-miR-3127-3p	7,16582418	-1,92895015	0,469453607	3,98E-05	0,000206604
hsa-miR-574-5p	9,40301845	-1,927340318	0,520970941	0,000216011	0,000861997
hsa-miR-6812-3p	8,41292692	-1,899724389	0,438407672	1,47E-05	8,71E-05
hsa-miR-1180-3p	433,830214	-1,89706984	0,254591645	9,23E-14	9,72E-12
hsa-miR-6749-3p	8,75881734	-1,895939937	0,481109637	8,12E-05	0,000373711
hsa-miR-6511b-3p	54,3299877	-1,884806255	0,375201919	5,08E-07	5,15E-06
hsa-miR-342-5p	74,6194964	-1,871817815	0,317983063	3,94E-09	9,37E-08
hsa-miR-3173-5p	262,130512	-1,871663311	0,358924582	1,84E-07	2,41E-06
hsa-miR-6803-3p	203,804481	-1,860389508	0,308792188	1,69E-09	5,09E-08
hsa-miR-3158-3p	94,4040875	-1,850648138	0,323213638	1,03E-08	2,06E-07
hsa-miR-6786-3p	22,9657782	-1,845241018	0,359152378	2,78E-07	3,04E-06
hsa-miR-6747-3p	107,259107	-1,836756599	0,377863962	1,17E-06	1,05E-05
hsa-miR-4732-3p	3235,33994	-1,812536072	0,33384368	5,66E-08	8,66E-07
hsa-miR-6881-3p	39,6605401	-1,811392624	0,34801863	1,94E-07	2,41E-06
hsa-miR-6894-3p	12,4957594	-1,804808581	0,360732955	5,64E-07	5,59E-06
hsa-miR-3168	45,8343555	-1,785625464	0,78063787	0,022172905	0,045535575
hsa_piR_020450/gb,	57,1056903	-1,769636514	0,440522409	5,89E-05	0,000290891
hsa_piR_008488/gb,	9,59978677	-1,761416144	0,472790661	0,000194872	0,000785082
hsa-miR-92b-5p	13,1973417	-1,760671826	0,370549461	2,02E-06	1,60E-05
hsa-miR-6734-5p	4,61102584	-1,760191063	0,578813954	0,002357697	0,006551752
hsa_piR_006465/gb,	19,6122861	-1,753457861	0,4108414	1,97E-05	0,000112989
hsa-miR-505-5p	154,346319	-1,735167316	0,331279402	1,63E-07	2,17E-06
hsa-miR-1229-3p	25,8948402	-1,704923421	0,305353441	2,36E-08	4,41E-07
hsa-miR-5694	6,2718628	-1,686309206	0,739261961	0,022544231	0,046073404
hsa-miR-3940-3p	61,1728503	-1,683035504	0,360627426	3,06E-06	2,26E-05
hsa-miR-766-3p	12,5873645	-1,672567919	0,314513064	1,05E-07	1,45E-06
hsa-miR-5006-3p	5,62737954	-1,666951573	0,466049375	0,000347866	0,001322048
hsa-miR-1227-3p	10,5832434	-1,640754083	0,461851213	0,000381502	0,00143404
hsa-miR-6754-3p	10,366469	-1,637063157	0,460261897	0,000375388	0,001417386
hsa_piR_002441/gb,	7,10369737	-1,629077051	0,553609058	0,003254173	0,00867093
hsa-miR-3615	4462,81864	-1,626198519	0,363784228	7,81E-06	4,92E-05
hsa-miR-3605-3p	317,911528	-1,608586217	0,333350059	1,40E-06	1,22E-05
hsa-miR-6833-3p	45,1093032	-1,602606905	0,320211477	5,59E-07	5,59E-06
hsa-miR-6877-5p	4,47357909	-1,60168387	0,591743721	0,006795178	0,016300684
hsa-miR-328-3p	625,641196	-1,600395636	0,380912035	2,65E-05	0,000144983
hsa-miR-130b-5p	322,985705	-1,596525106	0,29707992	7,70E-08	1,08E-06
hsa-miR-486-5p	587341,072	-1,594773584	0,321633935	7,11E-07	6,80E-06

hsa-miR-6799-3p	8,96029639	-1,5802283	0,383481229	3,78E-05	0,000198738
hsa-miR-1976	348,002626	-1,57582333	0,27084801	5,95E-09	1,22E-07
hsa-miR-1249-3p	18,3086035	-1,555812817	0,445613244	0,000480501	0,001746756
hsa-miR-6762-3p	4,37664465	-1,54755657	0,552906435	0,005126933	0,012847851
hsa-miR-3620-3p	14,1971524	-1,54707578	0,355263843	1,33E-05	8,01E-05
hsa-miR-1224-3p	7,1895306	-1,539639834	0,500084943	0,002078664	0,005912957
hsa-miR-6511a-3p	95,593003	-1,537052843	0,326184435	2,45E-06	1,88E-05
hsa-miR-486-3p	13996,3661	-1,518104823	0,311796922	1,12E-06	1,02E-05
hsa-miR-423-3p	3229,67318	-1,517793686	0,315307896	1,48E-06	1,27E-05
hsa-miR-6866-3p	8,975546	-1,475490029	0,467429503	0,001596177	0,004869497
hsa-miR-6884-3p	4,23205678	-1,472189146	0,595432912	0,013418352	0,029889557
hsa-miR-937-3p	35,8115386	-1,470023022	0,324734004	5,99E-06	3,94E-05
hsa-miR-6879-3p	10,7369573	-1,464854293	0,46367956	0,001582054	0,004843963
hsa-miR-92b-3p	1069,38858	-1,462365421	0,264238858	3,13E-08	5,60E-07
hsa-miR-7706	51,6712591	-1,455660076	0,279752293	1,96E-07	2,41E-06
hsa-miR-6515-3p	18,5884838	-1,454292811	0,416366242	0,000477947	0,001746756
hsa-miR-6748-3p	7,73449099	-1,438303776	0,4526156	0,001484175	0,00458261
hsa-miR-6802-3p	36,9269196	-1,432882405	0,336538477	2,07E-05	0,000116712
hsa-miR-1275	26,1423033	-1,431348069	0,400237982	0,000348568	0,001322048
hsa-miR-197-3p	722,88494	-1,405315594	0,314530821	7,90E-06	4,92E-05
hsa-miR-6734-3p	75,0438589	-1,403758144	0,287440864	1,04E-06	9,53E-06
hsa-miR-6861-3p	9,21506303	-1,394666511	0,438927624	0,001485811	0,00458261
hsa-miR-150-3p	7,67750269	-1,393909194	0,46943482	0,002984451	0,00802846
hsa-miR-181a-5p	570,56247	-1,393289159	0,301450945	3,80E-06	2,70E-05
hsa-miR-6780b-3p	7,88565403	-1,383865178	0,432750414	0,001384626	0,004350207
hsa-miR-484	18799,5913	-1,375109689	0,277604326	7,29E-07	6,90E-06
hsa-miR-659-5p	7,10793749	-1,364939292	0,441266541	0,0019799	0,005670327
hsa-miR-1294	50,874744	-1,351003132	0,226963251	2,64E-09	6,54E-08
hsa-miR-6782-3p	10,3406159	-1,348555509	0,357977671	0,000165122	0,000688281
hsa-miR-18a-3p	554,25337	-1,336104633	0,291167549	4,46E-06	3,10E-05
hsa-miR-6830-5p	6,9838689	-1,335403698	0,463615068	0,003971523	0,010321056
hsa-miR-324-3p	380,849428	-1,329411364	0,212607563	4,03E-10	1,36E-08
hsa-miR-6741-3p	31,9137732	-1,322845849	0,344374879	0,000122386	0,000542365
hsa-miR-6728-3p	19,8197318	-1,308602013	0,379093869	0,000556611	0,001977496
hsa-miR-6783-3p	21,1521866	-1,298828638	0,415951344	0,00179292	0,00526755
hsa-miR-18b-3p	106,770705	-1,27613254	0,339965238	0,000174233	0,000719139
hsa-miR-652-3p	586,728716	-1,273895035	0,265639259	1,62E-06	1,37E-05
hsa-miR-3150b-3p	8,62991683	-1,269444344	0,405539485	0,001746512	0,005196336
hsa-miR-6885-3p	11,6706131	-1,26170824	0,518012704	0,014864219	0,032677997
hsa-miR-4301	2014,90101	-1,25508071	0,292357841	1,76E-05	0,000102379
hsa-miR-193a-5p	41,7673113	-1,254909694	0,441508764	0,004478635	0,011427306
hsa-miR-92a-3p	169763,114	-1,240015124	0,24995478	7,01E-07	6,79E-06
hsa-miR-1273a	17,9536849	-1,234774306	0,329808122	0,000181173	0,000740524
hsa-miR-6817-3p	9,99502102	-1,232977398	0,395746889	0,001835894	0,005367439
hsa-miR-1270	33,6797461	-1,220400762	0,354918216	0,000584858	0,002060464
hsa-miR-4659b-3p	23,6648946	-1,212713575	0,3058796	7,35E-05	0,000346856
hsa-miR-3187-3p	14,1210649	-1,199359931	0,348100119	0,000570119	0,002016977
hsa_piR_008114/gb,	52,3714449	-1,190388162	0,281378778	2,33E-05	0,000129989
hsa-miR-1254	12,5420824	-1,186720851	0,30932808	0,000124827	0,000550285
hsa-miR-4510	9,13404561	-1,18174653	0,463882846	0,010849451	0,024689833

hsa-miR-501-3p	198,328871	-1,165469616	0,30496129	0,000132537	0,000581228
hsa-miR-877-3p	11,8851378	-1,163322464	0,425544441	0,006262188	0,01519528
hsa-miR-2110	266,853593	-1,142872197	0,311211574	0,000240347	0,000941267
hsa-miR-3200-5p	42,0037472	-1,140733619	0,432241893	0,008312416	0,019441818
hsa-miR-6859-5p	9,85890135	-1,137072473	0,395989898	0,004085697	0,010552627
hsa-miR-6514-3p	23,8257948	-1,13517522	0,333373347	0,000661357	0,002310634
hsa-miR-6758-3p	6,83608922	-1,126338439	0,479134614	0,018734278	0,039534491
hsa_piR_019912/gb/	277,605356	-1,120228925	0,372948327	0,002667087	0,007267596
hsa-miR-6775-3p	5,31846186	-1,107509832	0,465302005	0,017303446	0,037167095
hsa_piR_016735/gb/	74,0259003	-1,104798383	0,353162244	0,00175819	0,005212663
hsa_piR_016926/gb/	23,675738	-1,104190607	0,390896104	0,004731494	0,012036005
hsa-miR-664b-3p	13,9632756	-1,056562111	0,40974066	0,009919819	0,022883527
hsa-miR-1304-3p	70,3241697	-1,051435693	0,303058484	0,000521593	0,001868856
hsa-miR-629-3p	24,8469859	-1,046908699	0,275577286	0,000145301	0,000614792
hsa-miR-185-3p	127,015836	-1,042832448	0,222458423	2,76E-06	2,07E-05
hsa-miR-4725-3p	5,18700941	-1,027650902	0,417626968	0,013866976	0,030807371
hsa-miR-550a-5p	13,7052595	-1,026178526	0,296766452	0,000544473	0,001942568
hsa-miR-1468-5p	25,6237941	-1,019767113	0,347714025	0,003359469	0,008923258
hsa-miR-25-5p	27,840776	-1,016273465	0,306000215	0,000896464	0,003031418
hsa_piR_019752/gb/	9,93187921	-1,014045217	0,355692957	0,004359561	0,011157297
hsa-miR-296-5p	195,570735	-1,001839267	0,329237889	0,002343071	0,006532669
hsa-miR-6873-3p	23,9760428	-0,98893788	0,352189916	0,004985524	0,01253078
hsa-miR-5001-3p	28,570753	-0,979112463	0,303328615	0,001247054	0,004008924
hsa-miR-1260a	44,4317729	-0,919384308	0,247910702	0,000208466	0,000835849
hsa-miR-1260b	149,767521	-0,902856117	0,328991106	0,006063682	0,014861442
hsa-miR-150-5p	14254,7124	-0,875529832	0,338411353	0,009676607	0,022383799
hsa-miR-7641	10,3956466	-0,874525675	0,363663911	0,016182938	0,034938548
hsa-miR-574-3p	372,721899	-0,832311439	0,301918905	0,005838104	0,014415494
hsa-miR-532-3p	1286,64166	-0,829607181	0,275611808	0,002611989	0,007140567
hsa-miR-664a-3p	72,9299527	-0,829522833	0,356382998	0,019932494	0,041749153
hsa-miR-6806-3p	11,7329929	-0,819983824	0,346940233	0,018104553	0,038398069
hsa-miR-342-3p	4720,76163	-0,81258199	0,217367274	0,000185276	0,000753634
hsa-miR-942-5p	3491,06398	-0,799912402	0,298768908	0,00742045	0,017501454
hsa-let-7d-3p	557,660516	-0,799537612	0,299037509	0,007501876	0,017644078
hsa-miR-550a-3p	288,06359	-0,779241445	0,261883391	0,002924843	0,007893328
hsa-miR-6818-3p	55,4385445	-0,745295898	0,246733428	0,002522308	0,006917862
hsa-miR-181b-5p	82,1446897	-0,73615685	0,270288634	0,006457558	0,015545893
hsa-miR-744-5p	372,504279	-0,729846847	0,270714986	0,007017825	0,01659834
hsa-miR-1268b	26,9897023	-0,720146087	0,266524459	0,00689261	0,016447433
hsa-miR-6505-3p	28,0626797	-0,596261454	0,264800266	0,024338683	0,049144295
hsa-miR-576-5p	2059,76666	-0,59103732	0,249331961	0,017764833	0,037890964
hsa-miR-361-5p	891,496743	-0,576100306	0,245328864	0,018860452	0,039701252
hsa-miR-1255b-5p	67,7000739	-0,556187048	0,246112661	0,023828203	0,048345415
hsa_piR_000805/gb/	275,355955	-0,520693215	0,185050834	0,0048962	0,012343115
hsa-miR-191-5p	126971,133	-0,489979319	0,216111507	0,023374815	0,047540083
hsa-miR-625-3p	725,283594	-0,46218514	0,183172829	0,0116286	0,026250084
hsa-miR-625-5p	263,726469	0,339135853	0,145346496	0,019632812	0,041224009
hsa-miR-140-3p	11744,9649	0,439727188	0,176376946	0,012662949	0,028281705
hsa-miR-501-5p	164,159579	0,604432431	0,245826712	0,013941219	0,030890806
hsa-miR-25-3p	69516,9865	0,613707945	0,198923217	0,002034496	0,005806934

hsa-miR-29b-2-5p	31,1236997	0,619993124	0,267484981	0,020456853	0,042741116
hsa-miR-28-3p	372,540272	0,626487349	0,202090386	0,001934975	0,00557962
hsa-miR-1303	41,3844287	0,627253703	0,26209634	0,01670131	0,035965482
hsa-miR-421	354,322505	0,639526277	0,161224599	7,29E-05	0,000346856
hsa-miR-106b-3p	2432,30693	0,643239046	0,219592167	0,003397875	0,00899689
hsa-miR-192-5p	4168,16063	0,648188773	0,225635388	0,004069462	0,010543036
hsa-miR-2277-3p	56,8040806	0,654083756	0,275954124	0,017775452	0,037890964
hsa-miR-22-3p	5561,18244	0,660415178	0,272199919	0,015257282	0,033367875
hsa-miR-589-3p	23,5443217	0,660599336	0,285817325	0,020818269	0,043388571
hsa-miR-941	299,160074	0,70638697	0,227786777	0,001928106	0,00557962
hsa-miR-146a-5p	1032,19655	0,722386508	0,287748303	0,012056698	0,027071305
hsa-miR-451b	187,811326	0,741686525	0,235540121	0,001639	0,004964165
hsa-miR-548ay-3p	16,3481601	0,777980427	0,281527939	0,005719881	0,01417639
hsa-miR-15b-3p	1654,84222	0,780649195	0,238298568	0,00105315	0,003532878
hsa-miR-4325	15,871072	0,818695429	0,278090427	0,003240105	0,008660852
hsa-miR-142-5p	15112,2383	0,827671591	0,260987545	0,001517547	0,004663412
hsa-miR-10a-5p	96,5816694	0,829762187	0,343392693	0,015676325	0,034019242
hsa-miR-548ap-3p/5	25,6818492	0,852481486	0,369302378	0,020979325	0,043405729
hsa-miR-425-3p	245,204662	0,865683207	0,227037306	0,000137315	0,000594823
hsa-miR-130a-3p	640,471576	0,900925009	0,287104808	0,001701248	0,005079614
hsa-miR-3688-3p	44,1526123	0,913360231	0,299693727	0,002306419	0,006451842
hsa-miR-26a-2-3p	14,0479007	0,934456251	0,335848129	0,005396225	0,013482555
hsa_piR_001152/gb,	26,7963234	0,940073921	0,41816383	0,024569843	0,049492362
hsa-miR-7-1-3p	39,4131931	0,948015995	0,306080244	0,001953058	0,005612542
hsa-miR-3157-5p	17,753715	0,958288608	0,264499596	0,000291178	0,001129824
hsa-miR-181a-3p	34,1213491	0,968639204	0,381085567	0,011028665	0,025030015
hsa-miR-199a-5p	98,2912287	0,971038081	0,334052361	0,003650953	0,009546902
hsa-miR-1307-5p	19,954165	0,971781844	0,431049692	0,024167601	0,048916153
hsa-miR-23b-3p	1122,19567	1,010464457	0,255287317	7,55E-05	0,00035333
hsa-miR-6126	1671,03137	1,013020137	0,399185138	0,011157719	0,025254837
hsa-miR-215-5p	32,6387134	1,016965881	0,311419308	0,001092386	0,003631619
hsa-miR-30e-5p	1854,97203	1,021258567	0,316618305	0,001257442	0,004025727
hsa-miR-627-5p	25,9870721	1,029731726	0,32646497	0,001609456	0,004892283
hsa_piR_020326/gb,	25,39031	1,030097686	0,353871357	0,003603452	0,009452045
hsa-miR-127-3p	31,0773064	1,030412501	0,423632296	0,015002134	0,032895304
hsa-miR-1272	38,3535126	1,034202438	0,331079921	0,001785776	0,00526755
hsa-miR-628-5p	16,1421745	1,046544992	0,384281655	0,006461768	0,015545893
hsa_piR_004308/gb,	31,9192394	1,061803585	0,313953514	0,000719495	0,002503366
hsa-let-7i-3p	62,2053211	1,069427901	0,318428073	0,000783781	0,002704687
hsa-miR-99b-5p	218,50799	1,090482006	0,268135821	4,76E-05	0,00024312
hsa-miR-6071	20,0283726	1,106227624	0,35431815	0,001795471	0,00526755
hsa-let-7a-3p	42,8897328	1,106382314	0,299256837	0,000218077	0,000866135
hsa-miR-7-5p	538,381048	1,135103197	0,436885268	0,009372146	0,021739247
hsa-miR-4772-3p	32,5029079	1,141799798	0,405255773	0,004840222	0,012275502
hsa-miR-548au-5p/5	16,4339398	1,146398719	0,343999156	0,000860501	0,00292154
hsa-miR-30d-3p	6,46447684	1,156036271	0,450893599	0,010350986	0,023748039
hsa_piR_008112/gb,	535,253303	1,162181732	0,475935664	0,014610666	0,032204663
hsa-miR-769-5p	16,5090603	1,178868551	0,338988684	0,000505916	0,001820432
hsa_piR_001312/gb,	36,2514001	1,184379248	0,3017031	8,65E-05	0,000395821
hsa-miR-17-3p	27,1849367	1,189070521	0,412679728	0,003959885	0,010321056

hsa-miR-548o-3p	11,651429	1,198812695	0,320092709	0,000180247	0,000740331
hsa-miR-551a	5,27548129	1,208406887	0,538607893	0,024859797	0,049956919
hsa-miR-532-5p	544,286123	1,218213126	0,230138646	1,20E-07	1,63E-06
hsa-miR-432-5p	27,0145711	1,239042945	0,320713745	0,000111823	0,000500826
hsa-miR-103a-3p	20377,2855	1,250802512	0,311759187	6,02E-05	0,000294633
hsa-miR-223-3p	109936,347	1,303680283	0,236743542	3,66E-08	6,16E-07
hsa-miR-24-3p	2299,08697	1,318546513	0,203385039	8,99E-11	4,18E-09
hsa-miR-5196-5p	7,79451698	1,319425426	0,44099623	0,002772289	0,007505684
hsa-miR-103b	475,955424	1,325931124	0,330123524	5,91E-05	0,000290891
hsa-let-7g-5p	6846,71097	1,329873139	0,504853883	0,008434217	0,019672052
hsa-miR-340-5p	56,2465963	1,345601456	0,498926228	0,006996836	0,016595312
hsa-miR-548e-3p	7,2431051	1,358952212	0,447646973	0,002399241	0,00662348
hsa-miR-23a-3p	3793,20385	1,363565949	0,226893519	1,86E-09	5,40E-08
hsa-miR-145-5p	219,665418	1,364763048	0,294214769	3,51E-06	2,55E-05
hsa-miR-192-3p	12,4373436	1,364989474	0,385006194	0,000392062	0,001460691
hsa-miR-654-3p	41,4379943	1,374122844	0,536723787	0,010461048	0,023935333
hsa-miR-186-3p	6,51802056	1,374448658	0,447396397	0,00212555	0,006005749
hsa-miR-3692-5p	250,697422	1,378157039	0,305904581	6,63E-06	4,26E-05
hsa-miR-107	2229,1907	1,385334951	0,338770705	4,33E-05	0,000222143
hsa-miR-363-3p	2451,52363	1,407217091	0,419716178	0,000800035	0,002738332
hsa-miR-195-5p	40,8529718	1,423437794	0,406461811	0,000461743	0,00170521
hsa-miR-26a-5p	14795,6811	1,455603764	0,314524723	3,69E-06	2,66E-05
hsa_piR_000765/gb,	648,006764	1,457539825	0,531633738	0,006113628	0,014892341
hsa-miR-142-3p	10726,0641	1,467552114	0,420386619	0,000481292	0,001746756
hsa-miR-152-3p	66,5271414	1,471493125	0,291739925	4,56E-07	4,68E-06
hsa_piR_020496/gb,	463,164783	1,474464753	0,267046065	3,36E-08	5,78E-07
hsa-miR-335-5p	224,044428	1,480411902	0,343869635	1,67E-05	9,83E-05
hsa-miR-148b-3p	1550,65384	1,522138329	0,27812469	4,43E-08	7,04E-07
hsa-miR-223-5p	70,6272879	1,555260355	0,264948126	4,36E-09	9,91E-08
hsa_piR_001170/gb,	44,1221035	1,556967251	0,348589339	7,95E-06	4,92E-05
hsa-miR-643	5,74225629	1,598678624	0,492139551	0,001160456	0,003787226
hsa-miR-618	8,27909506	1,599930377	0,419968839	0,000139169	0,000594823
hsa-miR-30e-3p	718,12217	1,666170309	0,26531105	3,38E-10	1,19E-08
hsa-miR-1539	9,499072	1,702609731	0,392556223	1,44E-05	8,62E-05
hsa-miR-194-5p	2015,4677	1,727106931	0,235259908	2,12E-13	1,78E-11
hsa-miR-26b-5p	4933,58544	1,79855208	0,552215048	0,001126053	0,003718183
hsa-miR-146b-5p	207,36339	1,804554807	0,347112641	2,01E-07	2,41E-06
hsa-miR-140-5p	68,1797486	1,806846471	0,426255617	2,25E-05	0,000126097
hsa-miR-126-3p	4950,01872	1,815536132	0,771850919	0,018663491	0,03948407
hsa-miR-190a-5p	97,9618907	1,815639945	0,543559418	0,000836944	0,002853063
hsa-miR-20b-5p	380,871934	1,830694891	0,451874829	5,09E-05	0,000258316
hsa-miR-16-5p	248774,63	1,838555484	0,517796398	0,000384164	0,001437625
hsa-miR-96-5p	263,394082	1,838917701	0,578694441	0,001484479	0,00458261
hsa_piR_020365/gb,	11,2480811	1,861741345	0,495866947	0,000173675	0,000719139
hsa-miR-133a-3p	37,9122117	1,863967903	0,616240236	0,002488451	0,006847305
hsa-miR-570-3p	14,2709065	1,882185214	0,476290113	7,76E-05	0,000360887
hsa-miR-27a-3p	152,623416	1,890029892	0,394470393	1,66E-06	1,38E-05
hsa-miR-21-3p	3,54391308	1,896173036	0,684292595	0,005588486	0,013921614
hsa-miR-19b-3p	2895,65201	1,916820716	0,434620357	1,03E-05	6,29E-05
hsa-miR-21-5p	1166,88106	1,942113452	0,617093692	0,001648457	0,004974912

hsa_piR_004721/gb,	28,8242378	1,949902555	0,376509729	2,23E-07	2,56E-06
hsa-miR-15b-5p	14761,9771	1,956665083	0,473242971	3,56E-05	0,000188307
hsa-miR-369-5p	4,86872478	1,985027197	0,735992803	0,006995168	0,016595312
hsa-miR-29a-3p	728,966863	1,996173878	0,333554924	2,17E-09	5,54E-08
hsa_piR_019914/gb,	29,5169857	2,000832889	0,351498311	1,25E-08	2,45E-07
hsa-miR-27b-3p	195,782709	2,004755756	0,387099366	2,23E-07	2,56E-06
hsa-miR-660-5p	201,885028	2,007877827	0,417087199	1,48E-06	1,27E-05
hsa-let-7g-3p	2,25435249	2,065958402	0,8234276	0,012108241	0,027114731
hsa-miR-510-3p	32,374531	2,127346785	0,471243339	6,35E-06	4,15E-05
hsa-miR-126-5p	477,51721	2,162173882	0,880560289	0,014070738	0,031095962
hsa-miR-487b-3p	3,93813589	2,202224389	0,956131351	0,021263907	0,043882868
hsa-miR-15a-5p	2012,04748	2,206630856	0,748010246	0,003177731	0,008521176
hsa_piR_017716/gb,	90,0122437	2,228131815	0,371155772	1,93E-09	5,43E-08
hsa-miR-101-3p	678,305608	2,237711506	0,59237084	0,000158378	0,000663452
hsa-miR-301a-3p	7,20864999	2,249687286	0,556179992	5,23E-05	0,000263932
hsa-miR-374a-5p	211,815179	2,25346099	0,894705303	0,01178014	0,026521064
hsa-miR-3140-3p	2,78405676	2,282800534	0,832743704	0,006119656	0,014892341
hsa-miR-29b-3p	1940,73186	2,303574505	0,391505903	4,01E-09	9,37E-08
hsa-miR-6815-3p	2,4854975	2,3495145	0,885032644	0,007937409	0,018616431
hsa-miR-374b-5p	319,617	2,351287657	0,425630497	3,31E-08	5,78E-07
hsa_piR_020829/gb,	25,1836799	2,363367444	0,384582637	7,98E-10	2,59E-08
hsa_piR_017724/gb,	36,7801798	2,389827726	0,514779502	3,44E-06	2,52E-05
hsa-miR-548as-5p	1,65957847	2,457276936	1,01636018	0,01561798	0,033980204
hsa_piR_009294/gb,	2,57122148	2,458633159	0,902792502	0,006462069	0,015545893
hsa-miR-34a-5p	20,3311922	2,482102645	0,543292245	4,91E-06	3,33E-05
hsa-miR-3143	6,14628682	2,484400006	0,617819846	5,79E-05	0,000288439
hsa-miR-497-5p	12,6963668	2,508090729	0,878953306	0,004324092	0,01110026
hsa-miR-548e-5p	2,16999133	2,508404832	1,037370218	0,015604272	0,033980204
hsa-miR-135a-5p	4,71416808	2,527302931	0,787153342	0,001324181	0,004189992
hsa-miR-582-3p	4,8494345	2,577761539	0,701490549	0,000238137	0,000936968
hsa-miR-6069	3,33176028	2,612308238	0,775444861	0,000754999	0,002616086
hsa-miR-199a-3p	95,5511787	2,638739281	0,879580947	0,002699832	0,007333092
hsa-miR-29c-3p	819,32509	2,653859973	0,538942956	8,47E-07	7,84E-06
hsa-miR-381-3p	1,95254952	2,690424413	0,980516033	0,006071658	0,014861442
hsa-miR-19a-3p	167,774584	2,69514412	0,529478987	3,58E-07	3,77E-06
hsa-miR-5581-3p	1,59713033	2,805253363	1,164990138	0,016041799	0,034722865
hsa-miR-624-5p	15,531477	2,865431766	0,601457269	1,90E-06	1,54E-05
hsa-miR-590-5p	0,93828696	2,902949888	1,22729583	0,018014398	0,038303342
hsa-miR-6716-3p	2,89570197	2,924262591	0,929728838	0,001659248	0,004989597
hsa-miR-570-5p	2,16843274	2,926601001	0,911793987	0,001328655	0,004189992
hsa_piR_016945/gb,	3,44008891	2,932391216	0,874600107	0,000799879	0,002738332
hsa-miR-106a-3p	3,49741204	2,957366595	0,746076526	7,37E-05	0,000346856
hsa-miR-145-3p	5,48205162	2,971543899	0,627147397	2,16E-06	1,68E-05
hsa_piR_004309/gb,	4,9636922	2,986424459	0,81040219	0,0002286	0,000903669
hsa-miR-424-5p	48,3380408	3,026772798	0,606697771	6,07E-07	5,94E-06
hsa_piR_016677/gb,	5,13285476	3,040302884	0,831787474	0,000257038	0,001001971
hsa-miR-4785	1,06592673	3,044136111	1,318764984	0,020981154	0,043405729
hsa-miR-6780a-3p	1,07798215	3,047201113	1,325925713	0,021552183	0,044369042
hsa-miR-450b-5p	3,32912252	3,200417847	0,838089054	0,000134152	0,000585265
hsa-miR-4745-5p	1,21640458	3,235410134	1,423112741	0,022997618	0,046886184

hsa-miR-199b-5p	98,9866854	3,253269242	0,420487431	1,02E-14	1,43E-12
hsa-miR-450a-5p	2,05172012	3,256299539	1,000923748	0,001140753	0,003737408
hsa-miR-3667-3p	2,83597537	3,287130215	1,022576388	0,001306459	0,004151089
hsa-miR-4662a-5p	1,26958738	3,289961315	1,282567671	0,010313518	0,023726728
hsa-miR-3128	2,14153054	3,291397237	0,852582254	0,000113153	0,000504101
hsa-miR-4635	2,8699297	3,296555702	0,942949794	0,000472283	0,001736515
hsa-miR-5684	1,26144434	3,304194627	1,393853866	0,017761836	0,037890964
hsa-miR-23c	3,67752893	3,357846378	0,799196096	2,65E-05	0,000144983
hsa-miR-4676-3p	1,3442854	3,377734327	1,250154649	0,00689542	0,016447433
hsa_piR_020485/gb,	1,81566623	3,412058616	0,964565851	0,000404082	0,001498841
hsa_piR_002811/gb,	1,3662233	3,446251083	1,492737795	0,020961449	0,043405729
hsa_piR_020401/gb,	1,43454482	3,456366191	1,084825598	0,001442032	0,004513721
hsa-miR-3157-3p	1,41201313	3,466069025	1,356721757	0,010626766	0,02424861
hsa-miR-218-5p	1,44320157	3,47057098	1,115021096	0,001854718	0,005403711
hsa_piR_016742/gb,	3,95151843	3,470682767	0,885739737	8,91E-05	0,000401362
hsa-miR-143-3p	180,624387	3,472109872	0,443001775	4,59E-15	8,12E-13
hsa-miR-103a-2-5p	2,41498017	3,485607793	0,883871661	8,03E-05	0,000371397
hsa-miR-224-5p	29,1999398	3,501099042	0,672656589	1,94E-07	2,41E-06
hsa_piR_020499/gb,	1,94097234	3,527005603	1,123381252	0,001691592	0,005068757
hsa-miR-338-3p	29,4559393	3,535843843	0,484150273	2,81E-13	2,15E-11
hsa_piR_004307/gb,	28,393548	3,537399376	0,546910553	9,93E-11	4,18E-09
hsa_piR_019544/gb,	1,54580391	3,56544218	1,099695633	0,001186073	0,003855884
hsa_piR_004271/gb,	1,52527344	3,581061123	1,106795354	0,001214204	0,003932152
hsa-miR-933	1,54384185	3,583281399	1,001499951	0,000346346	0,001322048
hsa_piR_022296/gb,	1,5351762	3,596430203	1,114202481	0,001247432	0,004008924
hsa-miR-6892-5p	1,5591187	3,596833807	1,169056568	0,002093074	0,005933899
hsa-miR-3666	1,56320438	3,61631247	1,105739258	0,001073602	0,003587194
hsa-miR-23b-5p	1,56870725	3,625649324	1,01039166	0,000332764	0,001285264
hsa-miR-4769-3p	1,58958186	3,627620354	1,193840038	0,002376683	0,006582787
hsa-let-7f-2-3p	1,60557981	3,63275507	1,243933897	0,00349609	0,009227923
hsa_piR_001918/gb,	1,62001405	3,669063695	1,123834749	0,001095524	0,003631619
hsa-miR-4714-3p	1,68786868	3,748162322	1,16557629	0,001301195	0,004150024
hsa-miR-221-5p	7,06718643	3,767380513	0,74122873	3,72E-07	3,87E-06
hsa-miR-337-3p	3,03135312	3,776497013	0,948407321	6,84E-05	0,000332693
hsa_piR_009154/gb,	1,76455025	3,780446398	1,368159474	0,005724433	0,01417639
hsa_piR_018165/gb,	1,79591626	3,807335021	0,999107348	0,000138553	0,000594823
hsa-miR-1276	1,85919226	3,837257475	1,072305276	0,000345556	0,001322048
hsa_piR_004153/gb,	1,82342792	3,840158311	1,179670828	0,001132808	0,003725877
hsa_piR_016984/gb,	3,21305371	3,904849458	0,852124367	4,59E-06	3,16E-05
hsa-miR-1273g-3p	2,02637897	3,961777389	0,997071464	7,08E-05	0,000340889
hsa-miR-3202	2,02257523	3,975610206	0,965406543	3,82E-05	0,000199811
hsa-miR-5096	2,02319131	3,979703918	0,956133483	3,15E-05	0,000168976
hsa-miR-607	2,03518829	4,00142278	1,071266557	0,000187547	0,000759203
hsa-miR-143-5p	2,10647389	4,035934787	1,00063402	5,50E-05	0,000275559
hsa-miR-502-5p	2,10821293	4,060154723	1,020673721	6,95E-05	0,000336422
hsa_piR_015026/gb,	5,97937766	4,064453257	0,750683959	6,15E-08	9,00E-07
hsa-miR-379-5p	2,84764937	4,07859188	1,071819592	0,000141634	0,000602303
hsa_piR_011187/gb,	2,23625476	4,122989123	1,251750235	0,0009885	0,00332927
hsa-miR-98-3p	2,28480188	4,146281864	0,938417849	9,94E-06	6,11E-05
hsa_piR_005799/gb,	2,3259078	4,193397676	1,223457323	0,000609171	0,002137173

hsa-miR-3117-3p	2,30578652	4,196927881	0,985092041	2,04E-05	0,000116072
hsa_piR_008983/gb,	2,33438477	4,213336254	1,074278757	8,78E-05	0,000398213
hsa-miR-31-5p	2,41366568	4,235006709	0,921461205	4,31E-06	3,02E-05
hsa-miR-362-3p	2,3658222	4,252622283	0,938033625	5,80E-06	3,85E-05
hsa-miR-199b-3p	8,23009003	4,257252748	0,822256073	2,25E-07	2,56E-06
hsa-miR-10b-5p	2,47540657	4,278027553	0,912913145	2,78E-06	2,07E-05
hsa-miR-542-3p	2,41385599	4,294932309	0,94279297	5,23E-06	3,49E-05
hsa-miR-3120-3p	2,50588222	4,301071332	1,005868058	1,90E-05	0,000109748
hsa-miR-6513-5p	3,29703952	4,346157427	0,846324626	2,82E-07	3,04E-06
hsa-miR-363-5p	2,6053731	4,349318297	0,911134237	1,81E-06	1,48E-05
hsa-miR-374b-3p	2,56769224	4,355752672	0,942587093	3,82E-06	2,70E-05
hsa_piR_015249/gb,	2,60572072	4,357114338	0,839158598	2,08E-07	2,46E-06
hsa-miR-452-5p	2,58939609	4,372904259	1,04404196	2,81E-05	0,000152582
hsa_piR_005271/gb,	2,73743635	4,424764788	0,862673681	2,91E-07	3,10E-06
hsa_piR_001107/gb,	2,82872096	4,502130179	0,83307863	6,51E-08	9,29E-07
hsa-miR-7850-5p	3,0673616	4,577083094	0,840066936	5,08E-08	7,92E-07
hsa-miR-9-5p	3,05126032	4,57932559	1,02480826	7,88E-06	4,92E-05
hsa_piR_016970/gb,	3,11193033	4,614561609	0,97352085	2,14E-06	1,68E-05
hsa-miR-4721	3,12974839	4,645672724	0,892231645	1,92E-07	2,41E-06
hsa-miR-3688-5p	3,89113555	4,969296838	0,830264222	2,16E-09	5,54E-08
hsa-miR-15a-3p	5,38821371	5,417805832	0,840851373	1,17E-10	4,69E-09
hsa-miR-374a-3p	7,28750664	5,836875575	0,959223692	1,16E-09	3,63E-08
